# Magnitude of nonadherence to diet and exercise recommendations and associated factors among type 2 diabetes patients on treatment follow-up at Asella Referral and Teaching Hospital, Arsi, Ethiopia: A cross sectional study

**DOI:** 10.1371/journal.pone.0330576

**Published:** 2026-06-10

**Authors:** Nebyu Mekonen, Zewdu Hurisa, Enku Selamu, Tesfa G/meskel, Ayalneh Demissie

**Affiliations:** 1 Department of Internal Medicine, Arsi University, College of Health Sciences, Arsi, Ethiopia; 2 Department of Public Health, Cardiff University, Cardiff city, United Kingdom; 3 Department of pediatrics, Arsi University, College of Health Sciences, Arsi, Ethiopia; 4 Department of Public Health, Arsi University, College of Health Sciences, Arsi, Ethiopia; King Faisal University, SAUDI ARABIA

## Abstract

**Background:**

Type 2 diabetes mellitus is a major public health problem worldwide. Nonadherence to lifestyle modifications in individuals with type 2 diabetes poses a great problem.

**Objective:**

This study assessed magnitude and factors associated with nonadherence to diet and exercise.

**Methods:**

An institution-based cross-sectional study was conducted on 302 type 2 diabetic patients at Asella Referral and Teaching Hospital of Arsi University. The data were collected via structured questionnaire. Multivariate logistic regression was used to determine the effects of independent variables on nonadherence to diet and exercise recommendations.

**Results:**

Of the individuals in the study, 247(81.8%) patients did not follow the exercise recommendations, and 157(52%) patients did not follow the diet recommendations for type 2 diabetes. Duration since diagnosis > 5 years (AOR = 2.1, 95% CI [1.18–3.36]), doctors’ advice (AOR = 2.3, 95% CI [1.24–4.47]) and family history of DM (AOR = 4.3, 95% CI [2.55–7.29]) were independent determinants of nonadherence to dietary recommendations. Similarly, female sex (AOR = 3.6, 95% CI [1.60–8.60]), duration > 5 years since the diagnosis (AOR = 2.1, 95% CI [1.03–4.43]) and educational status attending only primary school (AOR = 3.33, 95% CI [1.33–8.34]) and only reading and writing or illiteracy (AOR = 6.7, 95% CI [1.86–24.29]) were independently associated with nonadherence to the exercise recommendation of type 2 diabetes patients.

**Conclusions:**

Overall, in our study, many patients were nonadherent to diet and exercise recommendations. Female patients and patients who attended primary school or less and > 5 years since diagnosis were more likely to be nonadherent to exercise. The determinants of diet nonadherence were a lack of doctors’ advice, > 5 years since diagnosis and a lack of family history.

## Introduction

Diabetes mellitus (DM) refers to a group of metabolic diseases characterized by hyperglycemia (high blood glucose) resulting from defect in insulin secretion, insulin action, or both. Chronic hyperglycemia in diabetes is associated with long-term damage, dysfunction and failure of various organs especially eyes, kidneys, nerves, heart, and blood vessels [1]. Type 2 diabetes mellitus (T2DM) is the most common form of diabetes in adults, accounting for 90% of all diabetic adults [2].

According to a population-based study performed in 81 different countries, 439 million adults are expected to be diagnosed with diabetes by 2030. [3]. In Subsaharan Africa, the incidence of Noncommunicable diseases (NCDs) is predicted to exceed that of infectious diseases by the year 2030 [4,5].

According to a systematic review and meta-analysis, the overall magnitude of T2DM in Ethiopia is approximately 5.95% for patients aged  ≥  40 years and 3.6% for patients aged less than 40 years [6].

Emerging evidence suggests that exercise training activates the expression of cellular antioxidant systems [7,8]. Therefore, regular and moderate exercise training can have antioxidant and anti-inflammatory systemic protective effects on type 2 diabetes patients [9].

In patients with established type 2 diabetes, physical activity improves glycemic control and reduces visceral adipose tissue and plasma triglyceride levels [10]. Physical activity also has a beneficial effect on glycemic control by increasing tissue sensitivity to insulin [11]. Healthy dietary habits, such as eating foods high in fiber and whole grain but low in fats, sugars, and carbohydrates, can help decrease the blood glucose level and subsequently reduce the amount of insulin needed [12].

American diabetic association (ADA) suggests that people with type 2 diabetes should perform at least 150 min per week of moderate to vigorous aerobic exercise for at least 3 days during the week, with no more than two consecutive days between bouts of aerobic activity [1].

Diets rich in whole grains, fruits, vegetables, legumes, and nuts; moderate in alcohol consumption; and low in refined grains, red or processed meats, and sugar-sweetened beverages have been shown to reduce the risk of diabetes and improve glycemic control and blood lipids in patients with diabetes. With an emphasis on overall diet quality, several dietary patterns can be tailored to personal and cultural food preferences and appropriate caloric needs for weight control and diabetes prevention and management [13].

Lifestyle modifications among patients with T2DM are effective if healthcare practitioners understand patients’ reasons for adherence and nonadherence to diets and exercise recommendations [14]. Certain studies indicate that adherence to a prescribed diet and regular exercise is important for both the prevention and control of patients with type 2 diabetes mellitus [15,16].

Adherence to prescribed lifestyle changes has also been shown to improve glucose levels, decrease blood pressure and correct lipid abnormalities, which are factors associated with the micro- and macrovascular complications of diabetes [17].

In ARTH, we have many patients who visit diabetic clinics with T2DM who are non-adherent to lifestyle modifications, such as exercise and dietary recommendations, despite interventions such as advice and educational posters in the clinic. The problem could be patient related factors or the effectiveness and magnitude of the interventions performed, which were not studied in ARTH.

Assessing adherence to diet and exercise recommendations in T2DM patients will help identify the magnitude and associated factors in local areas, enabling targeted interventions such as exercise programs and dietary interventions, and if modifiable factors such as a lack of advice from physicians and nursing staff are noted, we can intervene with teaching programs to aid in increasing overall adherence to lifestyle modifications for patients with T2DM.

The results of this study will help health care providers provide education and awareness on diet and exercise interventions targeting specific groups of patients in the management of DM. Thus, patients receive appropriate care that fits them.

Therefore, objective of the study is to determine the level of adherence of T2DM patients to diet and exercise recommendations and identify socio-demographic, clinical and behavioral factors influencing non-adherence to diet and exercise recommendations among patients with T2DM on follow up at ARTH diabetic clinic.

## Materials and methods

### Study design

An institution based cross-sectional study was conducted to assess the magnitude and associated factors of nonadherence to diet and exercise modifications among T2DM patients on treatment follow up at the ARTH diabetic clinic.

### Study setting

The study was conducted at Asella Referral and Teaching Hospital. The hospital is found in Asella, which is the seat of the East Arsi zone, Oromia. The town is located 175 km southeast of the capital Addis Ababa. The ARTH serves a catchment population of more than 3.5 million people from Oromia regional states in the country. The hospital provides inpatient and outpatient services. In addition, this ARTH medical college teaches under graduate and postgraduate medicine and other health science students.

The ARTH diabetic follow-up clinic has a total of 2,463 patients, 1,500 of whom have T2DM. The clinic has 3 nursing staff members. Medical residents and internists work in the clinic all week. The study was conducted from January 1 to January 30, 2025

### Participants

The source population is all T2DM patients on treatment follow up at ARTH diabetic clinic.

Patients with type 2 diabetes who attended the diabetic clinic at ARTH for regular follow-up visits were included in the study but patients who are acutely ill and can’t respond to questions; patients with impaired memory or who can’t respond to the questions and patients with disability were excluded from the study.

### Variables

The outcome variables were nonadherence to diet and exercise recommendations. The determinants considered in the study included demographic and clinical characteristics, and a number of behavioral variables. We examined the explanatory value of a number of socio-demographic variables included age, sex, marital status, children status, educational status, residence, monthly and income. Then we examined respondents’ clinical variables: duration of the disease, family history of diabetes, doctors’ advice and comorbidity. Lastly the study assessed behavioral variables: BMI, social support, cigarette smoking, Khat chewing and alcohol consumption.

### Data sources/ measurement

The data were collected face to face using interviewer administered questionnaire that developed after reviewing different literature on related topic [18,19] The questionnaire was first developed in English and translated to Afan Oromo (the regional state language) by language expert and re-translated back to English to check its consistency. Data were collected by two Afan Oromo-speaking trained nurses at the diabetic clinic and supervised by master-level public health professionals to ensure data quality and consistency. Training was given for data collectors and supervisors for one day by principal investigator about the objective of the study and data collection methods. Each interview was carried out face-to-face and lasted approximately 20 minutes. Before conducting the actual study, the questionnaire was pre-tested for validity and reliability on 5% of sample size in Adam Hospital Medical College which is located 75 kilometers away from the study hospital and modification of the data collection tool was made on the logical sequence of the questionnaire, its grammar and how to conduct interview. During the actual data collection, close supervision was made by the supervisors and principal investigator on a daily basis to check consistency and completeness of questionnaire.

Participants were classified nonadherent to exercise, if a patient does not engage in moderate intensity exercise for at least 150 minutes per week and at least three times per week. It can be mixed with strength exercise or 75 minutes of vigorous intensity exercise [1]. Nonadherent to diet, if a patient uses simple sugar and sweetened beverages and simple starch (pasta, rice, white bread, cookies or cake), does not eat fruits or vegetables at least three times per week [20]. Participants were classified as ‘Non-adherent’ if they failed to meet any single component of the criteria for exercise or diet mentioned above, and ‘Adherent’ only if they met all requirements.

Moderate alcohol intake was defined as one drink per day for female and 2 drink per day for male [21]. One drink equals 12 grams of ethanol which is equivalent to one bottle of bear, a glass of wine, [21] 40 ml areke, 350 ml tella and 200 ml tej [22].

### Study size

The minimum sample size was calculated using Cochran’s formula: n = Z^2^pq/d^2^ [12] at a prevalence (p) of 64.3% and 36% to nonadherence to diet and exercise recommendations based on a study performed in Jimma Medical Center, respectively [19]. By taking the higher prevalence to get the maximum sample size: p = 0.643 and q = 1–0.643 = 0.357 and d, the tolerated margin of error = 5% at the 95% confidence level [23].

Thus, (1.96)^2^ (0.643) (0.357)/0.05x0.05 = 352.73724864 ∼353 patients. Since the target population is less than 10,000, we used the correction formula nf = No/ 1 + No/N, where nf is the corrected sample size and N is an estimate of the population size during the study period which is 1500. The corrected sample size was nf = 353/1 + (353)/1500 = 286 and by adding a 10% contingency for non-responses, the final sample size was 315 patients. Systemic random sample was used to select the required number of T2DM patients, taking every fourth patient (1,500/315 ~ 4.7 based on a visiting sequence) using the diabetic clinic follow-up record as a sampling frame

### Statistical methods

The data were analyzed using stata version 25. Descriptive statistics such as percentage and frequency were computed, and the mean with standard deviation was used to summarize the continuous variables accordingly. Both bivariate and multivariable logistic regressions were performed to identify significant factors associated with adherence. Those variables with p-value < 0.25 in bivariate analysis were considered for multivariable logistic regression analysis to control the effect of confounding variables. Multicollinearity was checked among independent variables using variance inflation factors, and then the highest VIF between the independent variables was found to be 1.299, which was below cut -off points. The model fitness was checked by using the Homer-Lemeshow goodness of fit test and found to be p-value = 0.551.The strength of associations was expressed using adjusted odds ratios (AORs) with 95% confidence intervals, and the significance of associations was declared at a p-value of less than 0.05. Finally, the results are presented using charts and tables.

### Ethical considerations

Ethical approval was obtained from Arsi University Ethical Review Committee (A/CHS/RC/138/2024) and permission letter was obtained from ARTH before field activities started. After a detailed explanation of the purpose, benefits, confidentiality of the information, and voluntary nature of participation in the study we obtained written informed consent from all participated T2DM patients prior to their participation. Name and other personal identifiers were not recorded to maintain confidentiality.

### Inclusivity in global research

Additional information regarding the ethical, cultural, and scientific considerations specific to inclusivity in global research is included in the Supporting Information (S1 File).

## Results

### Sociodemographic characteristics of the respondents

Interviews with 302 of the 315 patients who were required were completed successfully (response rate of 96%). Among the participants, 171 (56.6%) were male. A total of 161 (53.3%) of them were aged above 50 years. Most are married 204 (67.5%) and the majority (91.7%) had children. A total of 55.3% resided in rural areas. The educational background of the participants revealed that 109 (36.1%) had attained only primary school education, 98(32.4%) had completed secondary school or above and 31.4% only read and write or illiterate, as shown in Table 1.

**Table 1:** Demographic characteristic of the participants in ARTH, 2025. (n = 302).

Variable	Category	Frequency	Percentage
Age	30-50	141	46.7
	Above 50	161	53.3
Sex	Female	131	43.4
	Male	171	56.6
Marital	Single	27	8.9
	Married	204	67.5
	Divorced	33	10.9
	Widowed	38	12.6
Children	No	25	8.3
	Yes	277	91.7
Residence	Rural	167	55.3
	Urban	135	44.7
Education	Secondary school and above	98	32.4
	Primary school	109	36.1
	Illiterate and Only read and write	95	31.4
Occupation	Daily laborer	10	3.3
	Employed	48	15.9
	Farmer	112	37.1
	Housewife	73	24.2
	Other	5	1.7
	Private business	46	15.2
	Unemployed(not working)	8	2.6
Income in ETB*	< 5000	113	37.4
	5000–10000	136	45
	> 10000	49	16.2

Note:

ETB* Monthly income in Birr

### Clinical and behavioral characteristics

Among the 302 participants, 54.6% had T2DM for more than 5 years. A total of 46% of the participants reported a family history of diabetes. A total of 52.3% of the participants utilized only oral hypoglycemic agents and 25.2% used only insulin, and the remaining 22.5% employed both. Twenty-one percent of the participants did not receive advice from health professionals regarding lifestyle modifications for T2DM. A total of 92.4% of the participants had social support. Only 14 (4.6%) and 12 (4%) patients chewed khat and smoked cigarettes, respectively, whereas 226 (74.8%) patients did not drink alcohol (Table 2).

**Table 2:** Clinical and behavioral characteristics of participants in ARTH, 2025. (n = 302).

Variable	Category	Frequency	Percentage
BMI	<18.5	26	8.6
	24.9 - 18.5	226	74.8
	29.9 - 24.9	50	16.6
Duration	> 5 years	165	54.6
	≤ 5years	137	45.4
Comorbidity	No	123	40.7
	Yes	179	59.3
Family history	No	163	54
	Yes	139	46
Medication	Oral hypoglycemic agent	158	52.3
	Insulin	76	25.2
	Oral hypoglycemic agent and insulin	68	22.5
Advice	No	65	21.5
	Yes	237	78.5
Support	No	23	7.6
	Yes	279	92.4
Alcohol	Above moderation	23	7.6
	In moderation	53	17.5
	No	226	74.8
Smoking	No	290	96
	Yes	12	4
Khat	No	288	95.4
	Yes	14	4.6

Among the participants, 179 (59.3%) had comorbidities (Table 2), with hypertension being the predominant condition in 155 (86.5%) and 67 patients (37.4%) having cardiac illness; 9 (5%) patients had asthma, one patient had stroke, and 17 (9%) patients had other comorbidities (Chronic obstructive lung disease, chronic lower back pain).

### Magnitude of nonadherence to diet and exercise recommendations

Among the participants 247 of them (81.8%) were not adherent to exercise recommendations, and 157 (52%) of the participants were non-adherent to dietary recommendation of T2DM.

### Reasons for nonadherence to the Recommended Lifestyle Modifications

**Fig 1:**
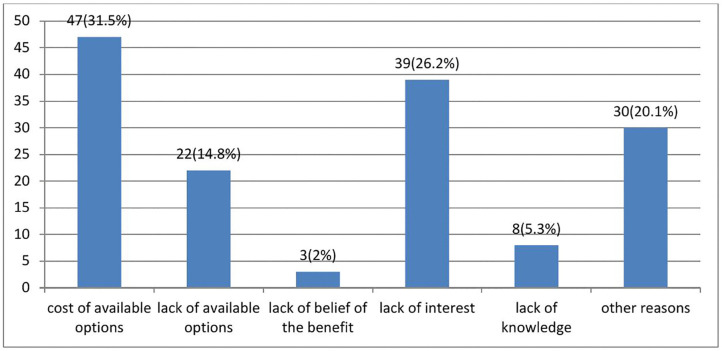
Reasons for nonadherence to dietary recommendations in ARTH, 2025. (n = 152).

**Fig 2:**
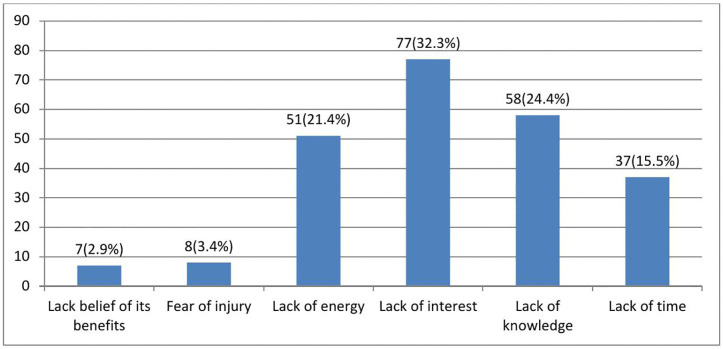
Reasons for nonadherence to exercise recommendation in ARTH, 2025. (n = 247).

The most common reason for nonadherence to dietary recommendations was cost of the recommended diet (47 patients). The most common reason for non-adherence to exercise was lack of interest (77 patients) followed by lack of knowledge (see Fig 1–2).

### Determinants of nonadherence to the Recommended Lifestyle Modifications

#### Determinants of nonadherence to exercise recommendations.

Binary logistic regression was performed to identify associations between the baseline characteristics of the patients and nonadherence to exercise recommendations. Sex, residential marital status, educational status, having children, and duration since diagnosis had p values < 0.25. After that, multivariate regression was performed to identify independent determinants of nonadherence to exercise recommendations, after which female sex (AOR = 3.6, 95% CI [1.60--8.60]), duration ≥ 5 years since diagnosis (AOR = 2.1, 95% CI [1.03--4.43]) and educational status attending only primary school (AOR = 3.33, 95% CI [1.33--8.34]), only reading and writing and being illiterate (AOR = 6.7, 95% CI [1.86--24.29]) were independently associated with nonadherence to exercise recommendations for T2DM patients (Table 3).

**Table 3:** Bivariable and multivariable analysis of determinants significantly associated with nonadherence to exercise in T2DM in ARTH, 2025. (n = 302).

Variable	Category	Adherence to Exercise		COR	AOR
		Adherent	Non-adherent		
Sex	Female Male	12 43	119 128	3.3(1.67-6.62)**	3.64(1.60-8.30)**
Residence	Rural Urban	14 41	153 94	4.7(2.46-9.21)*	2.49(0.96-4.44)
Marital status	Single Married Divorced Widowed	14 32 6 3	13 172 27 35	5.7(2.48-13.46)* 4.8(1.51-15.50)** 12.5(3.09-50.95)*	2.18(0.57-8.33) 1.34(0.26-6.72) 3.60(0.57-22.42)
Educational status	Secondary Primary Illiterate or only read and write	40 11 4	58 98 91	6.14(2.92-12.9)*** 15.69(5.33-46.14)***	3.33(1.33-8.34)* 6.72(1.86-24.29)**
Children	No Yes	9 46	16 231	2.8(1.17-6.78)*	1.02(0.21-4.89)
duration since diagnosis	> 5years ≤ 5 years	20 35	145 102	2.4(1.35-4.55)**	2.14(1.03-4.43)**

Note: *** very highly significant at p < 0.001

**highly significant at p < 0.01

* Significant at p < 0.05.

#### Determinants of non-adherence to dietary recommendation.

Binary logistic regression was performed to determine the associations between baseline characteristics and nonadherence to dietary recommendations, from which age, alcohol consumption, cigarette smoking history, duration since diagnosis, advice from healthcare professionals, and social support had p values < 0.25.

Furthermore, multivariate logistic regression was performed to identify independent determinants of nonadherence to the dietary recommendation of T2DM, and the results revealed that duration since diagnosis more than 5 years (AOR = 2.1, 95% CI [1.18–3.36]), lack of advice from health professionals (AOR = 2.3, 95% CI [1.24–4.47]) and having no family history of DM (AOR = 4.3, 95% CI [2.55–7.29]) were independent determinants of nonadherence to the dietary recommendations (Table 4).

**Table 4:** Bivariable and multivariable analysis of determinants significantly associated with nonadherence to diet in T2DM in ATRH, 2025. (n = 302).

Variable	Category	Non-adherence to diet		COR(95%CI)	AOR(95%CI)
		Adherent	Non-adherent		
Age(1)	30-50 Above 50	86 71	55 90	1.98(1.25- 3.13)**	1.53(0.85-2.77)
Alcohol	No In moderation Above moderation	129 20 8	97 33 15	2.19(1.18-4.05)* 2.49(1.01-6.11)*	2.19(1.07-4.48) 1.74(0.63-4.77)
Cigarette	No Yes	155 2	135 10	5.74(1.23-26.66)*	2.96(0.53-16.47)
Advice	Yes No	132 25	105 40	2.01(1.147-3.52)*	2.35(1.24-4.47)**
Family history	No Yes	58 99	105 40	4.48(2.75-7.29)***	4.30(2.55-7.29)**
Duration	>5 years ≤ 5years	70 87	95 50	2.36(1.48-3.76)***	2.14(1.18-3.36)*

Note: *** very highly significant at p < 0.001

**highly significant at p < 0.01

* Significant at p < 0.05.

## Discussions

### Magnitude of nonadherence to diet and exercise recommendations

In this study nonadherence to dietary recommendations was higher than study done in Nepal (41%) [24], Egypt (33%) [14] Jimma (36%) [19] Addis Ababa police hospital (46.3%) [25]. In contrast, this percentage was lower than what was reported in Tikur Anbessa Specialized Hospital in Addis Ababa (75.9%) [26]. Different cultural eating habits, different operational definitions on the term nonadherence to diet and geographical difference could have contributed to the observed differences.

Our participants’ adherence to exercise recommendation is almost similar to finding from a study in Yemen (84.8%) [27], Egypt (83%) [14] and higher than a study in Nepal (46%) [24], Addis Ababa (53.7%) [26] and Jimma (64.3%) [19] which can be in addition to cultural differences, due to different operational definition used in those studies in contrast to this study which used the current ADA guideline [1]. The nonadherence was also significantly higher than that reported in other studies at Addis Ababa police hospital (22.5%), where more than half of the participants were military [25] who could be adapted to performing exercise.

### Determinants of nonadherence to diet and exercise recommendations

Similar results were reported in other studies on the significance of lack of advice on diet and excersise [14,19,28] which could be due to a lack of knowledge of some of the recommendations. The other determinant was having no family history of DM which was not analyzed in other studies in respect to adherence to both diet and exercise [19,26] but this finding could be explained by the fact that having a family history could increase the familiarity with the dietary habits of diabetic patients. Furthermore, similar trend was reported in a study in Northwest Ethiopia [28] on significance of having DM for more than 5 years and nonadherence to diet, which could be explained by the fact that a longer duration of the disease could increase the likelihood of being fed up with following dietary recommendation.

This study was in line with the findings of a study in Jimma, [19] and Southwest Ethiopia [18], which showed that, females were more likely to be nonadherent to exercise explained by the culture of women staying at home with responsibility of raising children. The reason behind the association between not attending secondary school or above and exercise nonadherence, which was also evident in a study done in Jimma [19], could be related to a lack of knowledge of the recommended duration and benefit of exercise. Finally, patients with longer durations of illness (more than 5 years) might stop performing physical activity when they are accustomed to living with diabetes and get fed up with exercise.

### Innovation and Contributions of the Research

This study provides several key innovations that contribute to the existing body of literature on diabetes management in Ethiopia. First, while many studies focus on general adherence, this research specifically disentangles the unique barriers to diet versus exercise in the Arsi Zone, revealing that ‘lack of belief in the benefit’ and ‘cost of available options’ are more significant inhibitors than mere forgetfulness. Second, the study explores the intersection of traditional lifestyle factors—such as khat chewing and specific local dietary habits—with clinical outcomes in a major referral hub. By identifying these localized behavioral and socio-economic predictors, this research provides a tailored evidence base for clinicians at Asella Referral and Teaching Hospital to develop culturally-specific counseling interventions, moving beyond ‘one-size-fits-all’ diabetic education.

### Recommendations

Health care professionals should give emphasis for these groups of patients mentioned above when managing and advising patients on lifestyle modification. Particularly, as longer duration of DM was associated with poor adherence on both diet and exercise they should be given emphasis and their adherence followed and encouraged on a regular basis. Furthermore, there should be a support group for female patients with DM to encourage each other and mitigate the effect of gender role on their exercise adherence. Significant numbers of patients give reason of lack of knowledge as a cause of their nonadherence to exercise therefore, Physicians and nurses should provide specific and accurate guide on the recommended 150 minutes per week exercise recommendation during patients’ follow up visits.

It is good to have case control study in the future to show cause and effect relation on the outcome (nonadherence) and associated factors as cross sectional study doesn’t establish causality.

### Limitations

This research is not without its limitations. As we are only assessing patients’ responses to the question some patients may not report the true answer to avoid confrontation in addition to recall bias as well as social desirability bias. Despite our best efforts to account for a wide range of potential confounders, residual confounding might still exist. While the study provides valuable insights for the Asella region, it is possible that the findings may not be applicable to other settings with different medical practices, healthcare systems, or demographics.

## Supporting information

S1 File inclusivity in global research questionnaire. (DOCX)

S2 Appendix Survey Questionnaire. The English version of the interviewer-administered questionnaire used to collect socio-demographic, clinical, and behavioral data.
(DOCX)

S3 Data Dataset: Anonymized Raw Data. This file contains the fully anonymized primary data used for the analysis of nonadherence to diet and exercise among T2DM patients at Asella Referral and Teaching Hospital. Identifying information has been removed to ensure participant privacy.
(CSV)
