# Supplementary material for: Magnitude of nonadherence to diet and exercise recommendations and associated factors among type 2 diabetes patients on treatment follow-up at Asella Referral and Teaching Hospital, Arsi, Ethiopia: A cross sectional study
Source: PLoS One. 2026 Jun 10;21(6):e0330576. doi: 10.1371/journal.pone.0330576 (PMC13252749; doi:10.1371/journal.pone.0330576)
Supplement: S1 File — (DOCX) [file pone.0330576.s001.docx]

Inclusivity in global research

PLOS’ policy on inclusivity in global research aims to improve transparency in the reporting of research performed outside of researchers’ own country or community and ensures that PLOS publications reporting global research adhere to high standards for research ethics and authorship. Authors of relevant research articles may be asked to complete the questionnaire below, which outlines ethical, cultural, and scientific considerations specific to inclusivity in global research. This questionnaire may be requested when researchers have travelled to a different country to conduct research, if research uses samples collected in another country, research with Indigenous populations or their lands, or if research is on cultural artefacts. Researchers travelling to another country solely to use laboratory equipment will not normally be required to complete the questionnaire. However, the questionnaire can be requested at the journal’s discretion for any submission – if you have been requested to complete this questionnaire by the PLOS journal you submitted to, please do so.

Please complete the questionnaire below and include this as a Supporting Information file with your manuscript. Note that if your paper is accepted for publication, this checklist will be published with your article in the supporting information files. Please ensure that you reference the checklist in the main body of your manuscript. We suggest adding a subsection ‘Inclusivity in global research’ to your Methods section and adding the following sentence: “Additional information regarding the ethical, cultural, and scientific considerations specific to inclusivity in global research is included in the Supporting Information (SX Checklist)”

The questions have been designed to be applicable to a wide range of study types, and there are subsections for both human subjects research and non-human subjects research. If any of the questions are not relevant to your research please mark them as “N/A” as appropriate.

**Ethical considerations, permits and authorship**

*This section is applicable to all research types.*

Provide details as to who granted permissions and/or consent for the study to take place in the Methods section of your manuscript. This should include the names of **all** ethics boards, governmental organizations, community leaders or other bodies that provided approval for the study. If individuals provided approval refer to these people by their role or title but do not list their name(s).

Reported on page number: 9

If there were any deviations from the study protocol after approval was obtained please provide details of these changes in the Methods section of your manuscript.
Did this study involve local collaborators that are residents of the country where the research was conducted or members of the community studied? If you do not have any authors from said communities, please provide an explanation for this below. **Yes.** The study was led and conducted by researchers from **Arsi University, Ethiopia**, which is the primary institution in the region where the research was performed. The research team consists of residents of the country who are deeply integrated into the local academic and public health community. Furthermore, all data collectors were local residents who are fluent in the regional languages (Afan Oromo and Amharic), ensuring that the study was culturally and linguistically grounded in the community being studied

No deviations from the study protocol were made after ethical approval (A/CHS/RC/138/2024) was obtained. The study was conducted exactly as approved, with data collected via face-to-face interviews.

Reported on page number: 9

Everyone listed as an author should meet PLOS’ criteria for authorship and all individuals who meet these criteria should be included in the author byline, rather than the acknowledgements. For further information please see the journal’s Authorship Policy.

All individuals who contributed significantly to this research, including the design of the face-to-face data collection protocol for the 315 participants and the subsequent statistical analysis, are included in the author byline. We confirm that no local collaborators or researchers who meet the PLOS authorship criteria have been omitted or moved to the acknowledgements section

**Human subjects research (e.g. health research, medical research, cross-cultural psychology)**

Did you obtain written informed consent from a representative of the local community or region before the research took place? How did you establish who speaks for the community? Details of written informed consent obtained from study participants should be reported separately in the Methods section of your manuscript.

**Yes.** Before the study commenced, formal written permission and informed consent were obtained from the **District Health Office** and relevant local administrative leaders within the study region. We identified these representatives as the legitimate "voice" for the community because they hold the legal and administrative mandate for public health oversight and community welfare in this specific Ethiopian context. Their approval ensured the study was culturally appropriate and addressed local health priorities before we approached individual participants

How did members of the local community provide input on the aims of the research investigation, its methodology, and its anticipated outcome(s)?

Members of the local community and regional stakeholders provided input through several key channels: * **Preliminary Consultations:** Before finalizing the study aims, we held consultative meetings with District Health Office officials and community leaders to ensure the research addressed local public health priorities. * **Methodological Refinement:** Feedback from local health extension workers was used to refine the face-to-face interview process, ensuring that the terminology used in the translated questionnaires was culturally appropriate and easily understood by the 315 participants.

When engaging with the local community, how did you ensure that the informed consent documents and other materials could be understood by local stakeholders?

To ensure full comprehension by all stakeholders and participants, we implemented several strategies: * **Translation:** All informed consent documents and study instruments were translated from English into the local languages, **Afan Oromo and Amharic**, which are the primary languages spoken in the study area. * **Back-Translation:** The materials were back-translated by independent language experts at Arsi University to ensure the scientific and ethical nuances remained accurate. * **Oral Administration:** Because some participants may have had limited literacy, the consent forms were read aloud by trained data collectors in the local language. * **Witnessed Consent:** In cases where participants were unable to read or write, a thumbprint was obtained in the presence of an impartial witness to document voluntary participation. * **Training:** Data collectors received specific training on how to explain the study’s purpose, risks, and benefits in clear, non-technical terms to ensure participants could make a fully informed decision

Will the findings of the research be made available in an understandable format to stakeholders in the community where the study was conducted (e.g. via a presentation, summary report, copies of publications, etc.)? Please provide details of how this will be achieved.

**Yes.** We have a structured plan to share the results with local stakeholders to ensure the research has a practical impact on the community: * **Summary Reports:** A concise, non-technical summary of the key findings will be prepared in **Afan Oromo and Amharic**. These reports will be distributed to the **District Health Offices** and the administrative leaders who facilitated the face-to-face data collection for the 315 participants. * **Institutional Presentation:** A formal presentation of the results will be conducted at the **Arsi University College of Health Sciences**, inviting local health practitioners and policymakers to discuss the implications for regional health strategies. * **Community Briefing:** We will provide simplified feedback to the community leaders who assisted in the research to ensure that the 315 participants’ contributions are acknowledged and that the outcomes are understood by the local population. * **Open Access Publication:** As this manuscript is intended for a **PLOS journal**, the final peer-reviewed article will be published under an **Open Access** license, ensuring that researchers and health officials in Ethiopia can access the full scientific report without financial barriers.

**Non-human subjects research using specimens/ animals collected as part of the study, or those housed in archival collections. Examples include archaeology, paleontology, botany and zoology.**

Did the permission you obtained from a local authority to perform the study include an agreement on access to outputs and benefit sharing? This may include procedures to enable fair distribution of the benefits and resources arising from the research performed. Please include any details of Prior Informed Consent and Benefit Sharing Agreements obtained. These may be required by field-specific regulations, for example the Convention on Biological Diversity (CBD) and the associated Nagoya Protocol.

**N/A.** This study involves human subjects research (public health/epidemiology) and did not involve the collection or use of non-human specimens such as animals, archaeological artifacts, or botanical samples.

If the material used in your study was imported, please A) provide the year it was imported and B) indicate whether permits were obtained to import/export the materials used, C) provide details of any permits obtained. If this information is not available, please indicate this.

**N/A.** No physical materials, specimens, or artifacts were imported or exported for this study. The research consisted of primary data collection through face-to-face interviews with 315 participants within the local community, and all data was handled in accordance with the ethical approval provided by Arsi University (A/CHS/RC/138/2024)

If you used archival specimens, please state how the material used in your study was acquired by the institute it is held in and provide details of any permits obtained for the original excavations/ sample collection. If this information is not available, please indicate this.

**N/A.** This study did not utilize archival specimens, biological samples, or physical artifacts. The research is based on primary public health data collected directly from 315 human participants through face-to-face interviews. All data collection was conducted under the ethical approval of the **Arsi University Ethical Review Committee (A/CHS/RC/138/2024)**.

How was the potential cultural significance of the materials collected in your study to local communities considered in your research design? Were Indigenous peoples and/or local researchers and institutions involved with archaeological excavations / collection of specimens? If so, please provide a description of their involvement.

**N/A.** The study did not involve archaeological excavations or the collection of physical specimens. However, the research was led and conducted by local researchers from **Arsi University**, and all data collectors were residents of the local community.

If your manuscript includes photographs of human remains please indicate whether authors obtained permission from descendants or affiliated cultural communities to do so.

**N/A.** This manuscript does not include photographs of human remains. The study is a public health investigation involving living participants, and all data collection was performed with individual written informed consent and institutional ethical approval from **Arsi University (A/CHS/RC/138/2024**
