# Supplementary material for: Magnitude of nonadherence to diet and exercise recommendations and associated factors among type 2 diabetes patients on treatment follow-up at Asella Referral and Teaching Hospital, Arsi, Ethiopia: A cross sectional study
Source: PLoS One. 2026 Jun 10;21(6):e0330576. doi: 10.1371/journal.pone.0330576 (PMC13252749; doi:10.1371/journal.pone.0330576)
Supplement: S2 Appendix — The English version of the interviewer-administered questionnaire used to collect socio-demographic, clinical, and behavioral data. (DOCX) [file pone.0330576.s002.docx]

|  | Questionnaire for data collection | |
| --- | --- | --- |
|  | Are you willing to participate in this research Yes 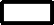 No 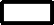 | |
| **1** | Age |  |
| 2 | Sex ((thick in box)) | 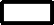 Female 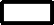 Male |
| 3 | Residency | 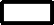 Rural 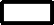 Urban |
| 4 | Marital status | 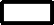 Single 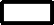 Divorced  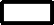 Married 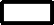 widowed |
| 5 | Do you have children | 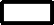 No 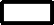 Yes |
| 6 | Educational status | 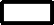 Illiterate or Read and write  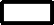 Primary school only  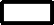 Secondary and above |
| 7 | Occupation | 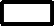 Daily laboror 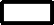 House wife 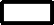 private business  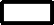Farmer 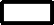 Employed 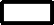 other |
| 8 | Monthly Income in ETB | 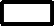 <5000 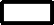 Above 10,000  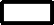 5000- 10,000 |
| 9 | BMI | 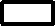 <18.5 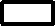 18.5-24.9 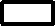 24.9-29.9 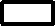 >29.9 |
| 10 | Duration since the diagnosis of DM | 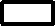≤ 5years 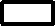 > 5 years |
| 11 | Any comorbidity | 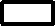 No 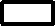 Yes, |
| 12 | If yes what is the comorbidity | 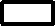Hypertention 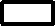Asthma  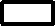Cardiac illness(Ischemic heart disease,Hypertensive heart disease,corpulmonale)  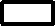 Stroke Others(chronic obstructive lung disease, chronic lower back pain ..) 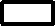 |
| 13 | Did he or she drink alcohol | 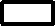 No 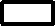 In moderation (2 drink per day for male and 1 drinks per day for female) 1 drink = 1 melekya Areke, 1 brile tej, 1 glass tella, 1 bottle of bear or 1 glass wine  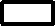 Above moderation |
| 14 | Do you chew khat | 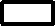 Yes 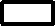 no |
| 15 | Do you smoke cigarette | 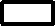Yes 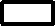 No |
| 16 | Does the patient receive any support from family or friends | 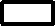Yes 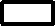 No |
| 17 | Was the patient advised on diet and exercise recommendation by the clinic stuff | 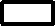 Yes 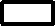 No |
| 18 | Do you have family history of DM | 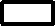 Yes 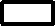No |
| 19 | Current medication | 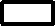 Insulin only 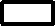Oral hypoglycemic agent only  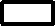 Both |
| 20 | Do you exercise 150 minutes per week moderate intensity exercise which can be mixed with strength exercise or 75 minutes vigorous intensity | 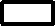Yes  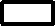No |
| 21 | If “no” to question 15 why? | 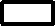Lack of belief of the benfit  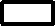Lack of energy 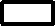Lack of time  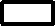Fear of injury 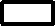Lack of knowledge  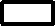Lack of interest Other 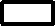 |
| 22 | Is the patient adherent to dietary recommendation of DM | Do you avoid sweetened beverages or added sugar  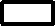 yes 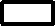 no  Do you eat fruit and vegetable ≥ 3 servings per week  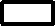 yes 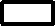 no  Do you avoid simple starch (pasta, white bread, white rice, cookies/cake)  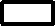 yes 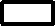 no |
| 23 | If “no” to question 16 why? | 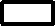 Cost of available options  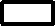 Lack of available questions  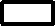 lack of interest 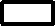 Lack of knowledge  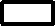 lack of belief of the benefit Others 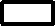 |
