# Supplementary material for: Magnitude of nonadherence to diet and exercise recommendations and associated factors among type 2 diabetes patients on treatment follow-up at Asella Referral and Teaching Hospital, Arsi, Ethiopia: A cross sectional study
Source: PLoS One. 2026 Jun 10;21(6):e0330576. doi: 10.1371/journal.pone.0330576 (PMC13252749; doi:10.1371/journal.pone.0330576)
Supplement: Attachment — Submitted filename: Response to Reviewers.docx [file pone.0330576.s004.docx]

**Point-by-Point Response to Reviewers**

**Manuscript ID:** PONE-D-25-38418 **Title:** Magnitude of nonadherence to diet and exercise recommendations and associated factors among type 2 diabetes patients on treatment follow-up at Asella Referral and Teaching Hospital, Arsi, Ethiopia: A cross-sectional study

**Journal Requirements:**

- **Data Anonymization:** We have replaced the previous data file with a fully anonymized version. All potential identifiers, including internal hospital IDs and specific location notes have been permanently deleted.
- **Table References and Formatting:** We have updated the text to ensure all tables (specifically Tables 3 and 4) are explicitly cited in the Results section. Furthermore, all tables have been reformatted to comply with **Vancouver style** requirements, ensuring clear headers, appropriate gridline usage, and standardized statistical reporting.
- **Inclusivity Questionnaire:** The "Inclusivity in Global Research" questionnaire has been completed and uploaded as a Supporting Information file.

**Reviewer #1:**

1. **Innovation Points:** A new subsection titled "Innovation and Contributions of the Research" has been added to the Discussion. It highlights the focus on specific barriers like "lack of belief in benefit" and the cultural context of the Arsi Zone.
2. **Repeating Results:** The Discussion has been revised to focus on the interpretation of associations (e.g., gender roles and health literacy) rather than restating the results.
3. **Future Research:** Recommendations for future research have been updated to align more logically with our findings regarding the duration of illness and lifestyle advice.
4. **STROBE Guidelines:** The manuscript has been audited and restructured to comply with the STROBE checklist for cross-sectional studies.

**Reviewer #2:**

1. **Introduction Length:** The Introduction has been streamlined, and background information has been moved to the Discussion where appropriate.
2. **Interview Structure:** We have included the survey questionnaire as **S1 Appendix**. We have also clarified that data were collected by two trained, Afan Oromo-speaking nurses and supervised by master-level public health professionals.
3. **Table/Figure Formatting:** Tables 3 and 4, and Figures 1 and 2, have been reformatted for alignment and corrected for typographical errors.
4. **International Comparisons:** We have added comparative context in the Discussion regarding adherence rates in other global regions to highlight the differences between developed and developing nations.
